# Gamification as a Pedagogical Model to Increase Motivation and Decrease Disruptive Behaviour in Physical Education

**DOI:** 10.3390/children9121931

**Published:** 2022-12-08

**Authors:** Marta Soriano-Pascual, Alberto Ferriz-Valero, Salvador García-Martínez, Salvador Baena-Morales

**Affiliations:** 1 Department of General and Specifics Didactics, University of Alicante, 03690 San Vicente del Raspeig, Spain; 2 EDUCAPHYS Research Group, Department of General and Specifics Didactics, University of Alicante, 03690 San Vicente del Raspeig, Spain; 3 Faculty of Education, International University of Valencia (VIU), 46002 Valencia, Spain

**Keywords:** innovation, self-determination, undesired behaviours, gamified approach, physical education, quality education

## Abstract

The application of gamified learning in physical education is becoming increasingly popular. The aim of this work was to compare the effects of gamification versus traditional methodology to check whether there were differences in the attitudes of the students. A quasi-experimental design study was carried out. The sample consisted of 66 students in Secondary Education. Three questionnaires, POSQ (Perception of Success), BPN (Basic Psychological Needs) and CCDEF (Disruptive Behaviour in Physical Education), were used in both groups before and after carrying out each proposal. Firstly, an independent samples Student’s t-test was performed. The results showed significant final differences in all variables except two: competence (*p* = 0.068) and aggressiveness (*p* = 0.136). Secondly, a paired samples t-test was performed. In this case, the control group showed a significant decrease in the variables task orientation (*p* = 0.004) and autonomy (*p* < 0.001). According to the experimental group, all variables showed significant differences (*p* < 0.05), except for two, competence (*p* = 0.223) and aggressiveness (*p* = 0.056). Therefore, it was concluded that, with the gamified learning, the students expressed higher levels of task orientation, all BPNs and lower levels of disruptive behaviours than the students who were subjected to the traditional methodology. This kind of intervention can help to improve the quality of education as set out in the SDGs through Quality Education.

## 1. Introduction

Currently, education is characterised in general terms by the absence of student motivation, lack of interest and lack of effort and discipline to achieve new knowledge and skills [1]. The reality of these discipline problems is increasingly frequent in classrooms [2], which lead to negative attitudes and expectations of students and teachers at the time of implementing the teaching–learning process [3]. In reference to the area of physical education, although it is presented by most students as a subject characterised by its enjoyment, in some students, a clear lack of motivation persists [4]. This fact occurs mainly in secondary education [5]. Along with this, as it is a predominantly practical subject and is characterised by constant interaction, it is not surprising that sometimes conflicting situations may arise [6]. Thus, authors such as Klomsten et al. [7] and Navarro-Patón et al. [8] indicate that disruptive behaviours may appear when there are cultural differences, a lack of skill and motor competence or the obsessive search for fighting among equals to win. These behaviours could become disruptive behaviours that make the teaching process difficult, such as aggressive behaviours (hitting or pushing); physically disruptive behaviours (smashing objects); socially disruptive behaviours (talking back out of turn or interrupting); authority-defying behaviours (refusing to comply with rules, defiant behaviours or offensive language) and self-disruptive behaviours (self-absorption or pride) [9].

In this regard, it is essential to consider that promoting motivation and enjoyment in sport in adolescents could facilitate their behavioural control [10]. To this aim, it is necessary to ensure that students adopt a goal-oriented perspective towards the task and not towards the ego [11]. Task orientation is understood as the pursuit of success through personal development [12]. Students continue participating and enjoying themselves, without the need for external rewards, as the real reward is individual achievement [13]. Effort is thus seen as a fundamental factor in achieving success and interest in learning, without concerns about failure and possible mistakes that are part of this learning process [14,15,16]. In contrast, the ego orientation brings with it comparison with peers and trying to surpass them in order to feel that they have achieved success [12,15]. These students maintain less autonomous motivation by considering learning as a means to achieve this feeling of superiority. In addition, students run the risk of avoiding making an effort in challenging tasks because they sense failure and thus shame [17]. Therefore, in these cases, in order to avoid negative emotions, they try in any way possible not to participate in the task at hand, or they try to do little or even lose interest in attending classes [18]. Therefore, teachers should consider incorporating innovative pedagogical approaches in their classes in order to become a strategy to guide students towards task orientation, foster their motivation and try to improve their attitude and discipline in the classroom. A good way to boost students’ motivation and deal with monotony and boredom is play [19]. Play is an attribute of a human being and has been present since the existence of the person, as a determinant means of learning, until senescence as a means of seeking enjoyment during rest time [20]. From there, a novel pedagogical approach emerges that is excelling in educational contexts. This is the term gamification.

The concept of gamification refers to the use of the characteristic elements of games in non-game environments [21]. Among the most important objectives presented in this type of active methodology is that of transforming the teaching–learning process into an attractive element for the students, and, ultimately, one that is much more effective to ensure the fulfilment of the didactic objectives [21]. In other words, gamification is understood to provoke in students “the same emotions and feelings that they feel with the games they are fond of, in order to engage them in the learning process” and, therefore, to represent in the classroom “magic, dreams, the feeling of overcoming, disconnection through fictional realities different from everyday ones, etc.” [22] (p. 8). Thus, effective gamification orients students towards intrinsic motivation, where they enjoy the process, as opposed to extrinsic motivation, where the aim is to obtain external rewards, or the student stops doing the process to avoid failure [22].

When making the decision to gamify educational contexts, it is necessary to consider the fundamental elements of games, which are the game elements themselves. Although there is no agreement among authors, Werbach and Hunter [23] propose classifying the main elements of games into three groups: (a) Dynamics: they establish the behaviours of the participants in each of the activities developed. This is the highest conceptual level and incorporates restrictive, emotional, narrative, relational, or progressive dynamics; (b) Mechanics: these correspond to the basic principles, i.e., the rules and functioning used to implement the dynamics of the games. It is the second level and includes challenges, rewards and incentives, levels and opportunities, competition and cooperation, feedback, etc.; (c) Components: they refer to the means that are available and the tools that are used. This is the basic level and includes achievements, badges or emblems, characters, points, gifts and unlocking content, leader boards or progress bars, etc. “Points (components) provide rewards (mechanics) and create a sense of progression (dynamics)” [24]. In this line, the proposal of activities through gamification improves the acquisition of skills such as problem solving, the creative component, control of emotions, autonomy, cooperation and communication. It also enables individuals to progress in a particular way through individualised feedback and enjoy learning through play [25].

For all these reasons, the aim of this study is to compare the results of a gamified proposal with a purely traditional proposal in the same teaching unit. In this way, to check if in either of the two methodologies there has been a significant change in the motivation of the students and thus in their behaviour.

## 2. Methods

### 2.1. Research Design

The design of this study is quasi-experimental and longitudinal. In it, two groups (experimental group and control group) are studied and evaluated with pre-test and post-test measures (after the four-week duration of the proposal). This is characteristic of the contexts in which the research is carried out in real classroom environments and the nature of the classroom itself (only handled by the gamification project intervention to increase motivation and improve student behaviour).

### 2.2. Participants

The sample selected to carry out this study was 66 students (age range between 13 and 16 years) enrolled in 2nd and 3rd year of Secondary Education (ESO) of a public school in the province of Alicante during the second quarter of the academic year 2021–2022. Of the total sample, 33 students (18 students of 2nd ESO and 15 students of 3rd ESO) were chosen to carry out the proposed gamified UD of orienteering “The Reconquest of the Outlaws”, while the remaining 33 students (17 students of 2nd ESO and 16 students of 3rd ESO) were chosen as a control group with a UD of traditional orientation. The main requirements to form part of the sample were based on the following: attending and participating in all of the sessions, completing the informed consent form for participation in the research and completing the initial and final questionnaires of the study anonymously and therefore, with the utmost sincerity (Table 1).

### 2.3. Evaluation and Data Collection Instruments

In order to collect data on the students’ experiences, three anonymous questionnaires were given to the 66 students participating in the study:

*(1) Success Perception Questionnaire (POSQ)* in its Spanish version [26] of the original questionnaire developed by Roberts and Balagué [27] and Roberts et al. [28]. This questionnaire is composed of two orthogonal principles designated as competitive orientation (determines the ego orientation of individuals) and mastery orientation (determines the task orientation of individuals). Both are composed of 6 items with closed-ended responses grouped on a Likert-type scale ranging from 1 (strongly disagree) to 10 (strongly agree) and begin with the following item: “In general in Physical Education I feel that I am successful when...”. Numerous authors [28,29] have demonstrated high internal consistency coefficients for the subscales that make up the POSQ in both the educational [30,31,32] and sporting [33,34] frameworks.

*(2) Basic Psychological Needs Questionnaire (BPN-PE)*, in the Spanish version [35]. This questionnaire is based on the Basic Psychological Needs in Exercise Scale [36] corroborated in the Spanish context by Moreno et al. [37]. It consists of 12 items assessing autonomy, competence and relatedness. The closed responses are collected on a Likert-type scale from 1 (strongly disagree) to 7 (strongly agree) and begin with the following heading: “In general, in physical education...”. The results obtained in the study of the Spanish version indicated that this questionnaire showed a 3-factor structure with high internal consistency. Thus, the subscales indicated the following internal consistencies (Cronbach’s alpha) for autonomy, competence and relatedness in the three samples, respectively: 0.84, 0.89, 0.85; 0.85, 0.87, 0.85 and 0.89, 0.92 and 0.89.

*(3) Questionnaire to Measure Disruptive Behaviours in Physical Education (CCDEF)*, adapted from the original abridged version of the Physical Education Classroom Instrument (PECI) by Krech et al. [38] and supported by the work of Kulinna et al. [39]. This questionnaire consists of 17 items to assess disruptive behaviours in PE students, namely: (a) aggressiveness, (b) irresponsibility and low engagement, (c) disobedience of rules, (d) disruptive to the classroom environment and (e) low personal self-control. Responses are collected on a Likert-type scale from 1 (never) to 5 (always) and begin with the following rubric: “Think about your own behaviour in PE class and tell us how much you agree with the following statements”. The internal consistency and validity values present in the satisfaction/enjoyment subscale were *α* = 0.78; composite reliability: *α* = 0.84; average extracted validity: *α* = 0.55.

### 2.4. Procedure

Parents/guardians, teachers and the participating students themselves were duly informed about the study and the objective of the study in order to carry out the fieldwork for this research. Thus, the questionnaires prior to the implementation of the study were explained in detail, and the participants were asked to be as honest as possible; their anonymous information was exclusively the subject of the study and was confidential. Regarding the context of the programme, on the one hand, the experimental group of the study followed a gamification methodology for the orientation didactic unit, which was inspired by the board role-playing game Bang: The Bullet and named “The reconquest of the outlaws! On the other hand, the control group of the study followed a purely traditional methodology for the same orienteering didactic unit, which was named “Initiation to orienteering”. Both programmes had a duration of 4 weeks, for a total of 7 physical education sessions (2 per week/50 min each). Thus, for the experimental group, a previous analysis was made of the mechanics of the game Bang: The Bullet, assessing its possibilities in the Physical Education classroom. To do this, a search was carried out on the official website of the game’s publisher daVinci Games: https://juegosdelamesaredonda.com/68-davinci-games and, in addition, the game was tested on several occasions and with different players to see first-hand its real functionality.

### 2.5. About the Game: “Bang: La Bala”

There is a great current interest among today’s youth in playing the fun and increasingly popular board role-playing game Bang: The Bullet. It was created by daVinci Games, the Italian publisher of board games and card games, and written by Emiliano Sciarra. Taking advantage of its appeal among young people and considering that many of the students play or have played it at some point in their free time, it was decided to include a didactic adaptation of this game in the Physical Education subject, emphasising three essential factors: interest and motivation towards sports practice, training in values and anticipation of the appearance of disruptive behaviour.

Bang: The Bullet is a card game for four to eight players set in the Wild West. In it, each player has a role to choose at random, which can be the following: sheriff, outlaw or renegade. Each role has its own objective in the game in order to win. Thus, the sheriff has to finish off the outlaws and the renegade; the sheriff is on the same side as the sheriff, so his mission is to protect the sheriff and finish off the outlaws and the renegade; the outlaws have to finish off the sheriff; and finally, the renegade has to finish off everyone, so he has to be the last player alive in the game. However, he must ensure that the sheriff does not die before the outlaws or the sheriff’s deputies, otherwise he will give the victory to the outlaws. As well as the role, each player is a character based on the Western movies. Each of them has a special ability unique to the course of the game and a number of life points represented by bullets. This is reflected on the character’s own card. To find out how many lives the character has left before being eliminated from the game, there is one more card, which shows the number of lives (with bullets) remaining for each cowboy.

Once each player has his or her cards, the game can begin.

Each player can only hold as many cards as he/she has lives. So, for example, if a player starts with 4 lives, he/she cannot hold more than four cards.

Each turn, the player draws two cards from the deck and, if it were for the example above, they would be added to the player’s four cards. The player can play as many times as he/she wants, as long as the number of cards left in his/her hands before his/her turn is equal to or less than the number of lives he/she has left. There are many types of cards: cards to skip the turn, cards to steal from another player, cards that add lives, cards to force a discard, etc., in any case, they are cards that, when played, can harm others or benefit one’s own character. However, in order to take a life from another player, which is the ultimate goal, it is necessary to play the mythical card of this game, the bang card. This card, which acts as a shot and can only be used once per turn, can take a life, as long as the attacked player does not have a card called “failed”, which works to cancel or dodge the shot. In addition, it is worth mentioning a card called duel, whereby playing it, a player is challenged to a duel and in turn they must throw a bang card. In this case, failed cards cannot be used, and the player who has no more bang cards among his/her cards loses the duel and loses a life.

The game, which lasts approximately 20 min, will end when one of the roles achieves his or her goal.

### 2.6. Common Elements of the Game and the Orienteering Sport

Then, the similarities of the game Bang: The Bullet were compared with the functionality and internal structure of the orienteering sport in order to interpret the way in which this game would fit into the possible orienteering activities.

In this sense, it was chosen to introduce this gamified proposal to the block of activities adapted to the environment and, more specifically, to the didactic unit of orienteering, as it often goes unnoticed and is not taught in the classroom [40]. In many cases, this is due to insufficient teacher training or capacity to develop engaging activities related in this case to guidance [41]. Gamification of the guidance DU is a good way to turn its activities into a fun and enjoyable situation for both students and teachers [42]. In addition to this, the adaptation of the game Bang: The Bullet presents ideal characteristics to be worked on in the aforementioned content block. This proposal is designed to be worked on in small co-educational groups that favour relationships between equals and the sense of belonging to a group. Participants have to overcome “challenges” as a team to unlock levels and obtain common rewards. Therefore, what better option than to work on orienteering in small groups where students work cooperatively and together learn to make the best decisions in the fun search for beacons.

### 2.7. Development of the Teaching Proposal

Once an in-depth analysis of the game had been carried out and after assessing its possibilities as part of the content block of activities adapted to the environment, a Didactic Unit of orienteering was designed, named “The Reconquest of the Outlaws” and for which current legislation was taken into account, both the Organic Law 8/2013 of December 9 for the Improvement of Educational Quality, and Decree 87/2015, of June 5 establishing the curriculum and developing the general organisation of Compulsory Secondary Education and Baccalaureate in the Valencian Community.

The gamified proposal “Watch where you’re going, outlaw” included mechanics widely used in games such as levels, which progressed according to the increasing complexity that manifested itself as the DU progressed, with the total number of levels designed being four: beginner, intermediate, advanced and expert. Along with these, there was a team level called “Team DNA”, referring to the ability as a team to achieve adequate communication, organisation and effectiveness in different tests. If they achieved each and every one of these objectives, they reached the maximum allocation in team DNA called “Champion DNA”. In order to pass a level and move on to the next one, the students had to pass a series of activities, considered as acceptable for their stage, with which they obtained a series of points (experience points, which were recorded in a classification table or ranking visible to the whole class and compensable, in the form of cardboard coins) that led them to reach a higher level in the classification table, as well as to obtain different achievements with a series of rewards for subsequent activities or in the final evaluation (final duel between the sheriff and the outlaws). In the same way that the levels brought a higher complexity to the progress of the DU, the achievements, in the form of a card, also carried a higher reward in accordance with the level of complexity at which they were found. Rewards were usually fixed and random, although there were also social rewards that generously helped peers in need. When students wanted to make use of the coins or achievements acquired at the time, they had to return the corresponding coin or physical card to the teacher.

As for the dynamics, the most used were mainly the relational dynamics, the dynamics of progression and the narrative dynamics, with the aim of getting the pupils to interact and perceive progress in the acquisition of their skills through the exhibition of challenges to be solved and through an optimal narration of the approach. In addition, and in order to ensure that the motivation of the participants would last, it was chosen to use a competition mechanics by making use of the leader board that involved a comparison between groups and individuals. However, following the recommendation of Teixes [43], a proportion between competition and cooperation was sought in any case.

Finally, the group that managed to reach the highest level, corresponding to expert + champion DNA, would receive a virtual diploma for each student member, considering their continuous effort throughout the course of the unit. In annex 2, the design of this diploma is attached as a final random reward that the students could not observe until the end, but that they knew existed.

At the same time as it was being made, a portfolio was created to show the adaptation of the fundamental pieces of the game Bang: The Bullet. This portfolio was created with the intention of making the participants’ achievements and progress more evident to everyone involved in the teaching–learning process [44], leading to a greater predisposition on the part of the students in this process, by generating a feeling of personal achievement. The aim was therefore to ensure meaningful learning where the student was aware of being at all times the protagonist of the progress he or she was experiencing in his or her own learning [45]. Annex 3 shows the portfolio of evidence named “Outlaw Album”, which contains everything necessary to carry out this gamified orienteering DU.

### 2.8. Statistical Analysis

SPSS statistical software version 24.0.0 was used to perform all statistical analyses. Basic inferential statistics (mean and standard deviation) were calculated. The Shapiro–Wilk normality test was performed, obtaining normal distributions in all cases (*p* > 0.05). Student’s t-test (paired) was used to verify the within-group effect of the intervention (pre-test vs. post-test). The dependent variables had two domains in goal orientation, three domains in basic psychological needs and five in disruptive behaviours. Finally, a 95% confidence interval was calculated for the differences and the significance value was determined at *p* < 0.05.

## 3. Results

### 3.1. Initial Sample Equivalence

As shown in Table 2, the assessment of students in both groups in the pre-test was similar. This statement is given by the scores of the different variables, which did not show significant differences except for one, task orientation (*p* < 0.001). This means that the two groups started from an analogous level of perception regarding the different items of the three questionnaires.

### 3.2. Final Comparison between Self-Control Groups

Table 3 shows the results of the post-test, which indicate that, after a 4-week intervention, there were significant differences between groups in all of the variables studied (*p* < 0.05), with the exception of the competence variable (*p* = 0.068) and aggressiveness (*p* = 0.136), where no significant differences were found.

### 3.3. Intra-Group Comparison

Table 4 shows the results extracted from the analysis of the related samples Student’s t-test, where it can be seen from the outset that there was a greater score differentiation between the tests of the experimental group than between those of the control group. In a detailed analysis of the results, it can be seen that, on the one hand, in the control group there were significant differences between both tests only in two variables: task orientation (*p* = 0.004), which was scored as 0.66 points less in the post-test mean, and the autonomy variable (*p* < 0.001), whose mean score in the post-test decreased by more than one point with respect to the pre-test. On the other hand, in the experimental group, practically the opposite occurred, as for all of the variables, the results showed significant differences (*p* < 0.001) with the exception of two, competence (*p* = 0.22) and aggressiveness (*p* = 0.056), the latter being a variable with a tendency to be significant as well. Thus, the most notable scores were obtained for the mean of the autonomy variable (3.79 ± 1.26 to 5.88 ± 0.68) and the mean of the ego orientation variable (7.17 ± 1.79 to 5.09 ± 1.47).

## 4. Discussion

The main objective of this study was to identify the impact generated by a gamified proposal on self-determination and the absence of motivation in secondary school students in order to try to modify undesired behaviours in the classroom. Through the subject of physical education, it is possible to merge motor learning with active methodologies and increase the motivation by using innovative teaching techniques supported by new methodologies such as gamification [46]. In this sense, the gamified proposal carried out in this research tended to promote the development of cooperation and group cohesion, to undertake new group challenges and to contemplate different characters in each group with different roles. Thus, cooperative work is promoted [47] and, consequently, undesired behaviours in the PE classroom are simplified [48]. This is important to bear in mind since, as Macazaga et al. [49] state, the tendency towards undesirable behaviour during PE sessions is more common than in other subjects, where students limit themselves to listening to the teacher and completing their tasks, remaining seated at all times.

In this line, the results extracted from this study indicate that, although the use of gamification as an innovative pedagogical model has been based mainly on rewards for work done [50] and sanctions for undesirable behaviour, the levels of intrinsic motivation of students increased significantly in the experimental group, who experienced the gamified proposal. In this group, the proposal had typical game elements such as an engaging narrative, avatars, badges, points, or special skills. These elements, which are characteristic of gamification, seem to contribute to student motivation, leading to greater individual initiative towards the value of effort and increased performance in PE tasks [51]. This suggests that, possibly, the effort to achieve rewards together with cooperative work has an indirect impact on intrinsic motivation or simply increases such motivation immediately [52].

Following the line of published research, the results obtained from the present study are similar to those of other authors who have supported the use of gamification as part of the subject of physical education. Yıldırım and Şen [53] showed that gamification has a moderately positive effect on student achievement, adding 7.2% positive value to academic achievement. Other authors such as Navarro-Ardoy et al. [54] point out that this type of methodology encourages increased commitment and participation, thus reducing absenteeism. This last statement could be seen in this intervention exactly in item 14, “I skip PE classes”, of the questionnaire to measure disruptive behaviour in PE (CCDEF), where students 3, 4 and 18 and students 8 and 23 scored it with a 1 (never) after having carried out the gamified proposal. In addition to this, the teacher and tutor of these students commented the following: “this particular student has always missed PE class on Thursdays without justified reasons and, since the start of the gamified PE unit, he has attended each and every one of the sessions showing, in addition, a great participative attitude”. Following the demonstrations of various authors, gamification also produces an increase in levels of autonomy, responsibility, motivation and academic performance [55,56,57]. Furthermore, the idea put forward by Llorens-Largo et al. [58] is reaffirmed, where they accredit that the secret ingredient that convincing gamification into a special experience is fun. Other authors [59] point out their relation to commitment, especially to the commitment to learning or improvements in motor competence.

These benefits, added to the effect of an increase in cooperative work [5,19,60,61,62,63,64,65] and class climate [19,62,66,67], would cause students to be more collaborative and, consequently, less aggressive, achieving the set of competences framed in each block of content. The statement on the reduction of aggressiveness has been shown in the results of the post-test of the experimental group with a tendency to significance (*p* = 0.056). In other words, the students scored lower on the aggression variable after the gamified intervention. This means that, if the participants in the experimental group showed lower levels on this variable in the classroom, it is speculative that, if the sample were larger, the post-intervention results would end up being significant with respect to the initial results and, therefore, possibly deal with this pattern of aggressive activity in the classroom.

## 5. Conclusions

Gamification in the physical education classroom counteracts the apathy and indifference of the students, detecting an increase in the levels of task orientation, autonomy and relationships and a decrease in the levels of ego orientation and the majority of disruptive behaviour variables. From the results obtained, it can be concluded that a physical education teacher who includes this innovative pedagogical model will provoke an optimal climate where the goal perspective is oriented towards approximation/mastery, which will result in students being involved in their learning, predisposed to prosper in their competences and achieving satisfaction for their personal achievement. With the use of gamification, students positively evaluate the social relationships created in the classroom, thus creating an optimal environment for a conducive teaching–learning process.. Thus, and by way of a final conclusion, it should be noted that gamification is currently one of the most interesting methodologies for achieving a high degree of student commitment, as well as students’ success and a possible flow state in which a student is immersed and focused on something specific and participating and enjoying the process.

## 6. Limitations and Future Perspectives

Firstly, one of the most important limitations found was the presence of the main author as a teacher, who delivered the intervention and was not blind to the conditions.

Secondly, it is necessary to increase the sample of students in different educational contexts. Another limitation is the lack of qualitative measures, which would be useful to acquire deeper conclusions.

For this reason, as future lines of research in physical education, it is proposed to continue with the work developed as well as deepen into more specific aspects of the subject, using different educational courses or stages (e.g., elementary students).

## Figures and Tables

**Table 1:** Initial and final sample according to the sample selection requirements.

Group	Course	Initial Sample	Girls	Boys	Excluded	Total
CON	2nd ESO	22	11	6	5	17
	3rd ESO	23	7	9	7	16
EXP	2nd ESO	21	9	9	3	18
	3rd ESO	18	6	9	3	15
	Total	86	33	33	18	66

CON = Control; EXP = Experimental group.

**Table 2:** Initial results of the independent samples Student’s t-test.

	Control	Experimental Group		
	M ± DS	M ± DS	t	Sig.
Goal orientation				
EGO	7.74 ± 1.61	7.17 ± 1.79	−1.351	0.181
TASK	8.18 ± 1.20	7.29 ± 0.83	−3.476	<0.001
Basic Psychological Needs				
AUTM	3.78 ± 1.56	3.79 ± 1.26	0.022	0.983
COMP	4.40 ± 1.06	4.46 ± 1.28	0.210	0.835
RL	4.73 ± 1.33	4.53 ± 1.04	−0.696	0.489
Disruptive behaviours				
AG	1.68 ± 0.58	1.67 ± 0.55	−0.108	0.914
IBC	2.32 ± 0.66	2.41 ± 0.63	0.574	0.568
DESO	2.20 ± 0.68	2.36 ± 0.62	0.946	0.347
PERT	2.17 ± 0.66	2.09 ± 0.59	−0.544	0.588
AUTC	1.59 ± 0.51	1.60 ± 0.56	0.077	0.939

AUTM = autonomy; COMP = competence; RL = relationship; AG = aggressiveness; IBC = irresponsibility and low commitment; DESO = disobedience; PERT = disruptive; AUTC = self-control.

**Table 3:** Final results of the independent samples Student’s t-test.

	Control	Experimental Group		
	M ± DS	M ± DS	t	Sig.
Goal orientation				
EGO	7.46 ± 0.94	5.09 ± 1.47	−7.820	<0.001
TASK	7.52 ± 0.66	8.81 ± 0.95	6.443	<0.001
Basic psychological needs				
AUTM	2.54 ± 0.74	5.88 ± 0.68	19.109	<0.001
COMP	4.30 ± 1.10	4.80 ± 1.09	1.858	0.068
RL	4.45 ± 1.07	5.52 ± 1.08	4.036	<0.001
Disruptive behaviours				
AG	1.62 ± 0.72	1.39 ± 0.48	−1.511	0.136
IBC	2.36 ± 0.85	1.70 ± 0.64	−3.944	<0.001
DESO	2.17 ± 0.49	1.53 ± 0.71	−4.250	<0.001
PERT	2.02 ± 0.74	1.43 ± 0.44	−3.944	<0.001
AUTC	1.60 ± 0.48	1.20 ± 0.32	−3.932	<0.001

AUTM = autonomy; COMP = competence; RL = relationship; AG = aggressiveness; IBC = irresponsibility and low commitment; DESO = disobedience; PERT = disruptive; AUTC = self-control.

**Table 4:** Intra-group differences (pre-test vs. post-test) by intervention group.

Group	Control			Experimental Group		
Variable	Pre-Test M ± DS	Post-Test M ± DS	t	Pre-Test M ± DS	Post-Test M ± DS	t
Goal orientation						
EGO	7.74 ± 1.61	7.46 ± 0.94	1.19	7.17 ± 1.79	5.09 ± 1.47	4.73 **
TASK	8.18 ± 1.20	7.52 ± 0.66	3.13 *	7.29 ± 0.83	8.81 ± 0.95	−6.05 **
Basic psychological needs						
AUTM	3.78 ± 1.56	2.54 ± 0.74	4.65 **	3.79 ± 1.26	5.88 ± 0.68	−8.10 **
COMP	4.40 ± 1.06	4.30 ± 1.10	0.42	4.46 ± 1.28	4.80 ± 1.09	−1.24
RL	4.73 ± 1.33	4.45 ± 1.07	0.96	4.53 ± 1.04	5.52 ± 1.08	−3.60 **
Disruptive behaviours						
AG	1.68 ± 0.58	1.62 ± 0.72	0.36	1.67 ± 0.55	1.39 ± 0.48	1.98
IBC	2.32 ± 0.66	2.36 ± 0.85	−0.19	2.41 ± 0.63	1.70 ± 0.64	4.11 **
DESO	2.20 ± 0.68	2.17 ± 0.49	0.26	2.36 ± 0.62	1.53 ± 0.71	6.31 **
PERT	2.17 ± 0.66	2.02 ± 0.74	0.95	2.09 ± 0.59	1.43 ± 0.44	4.87 **
AUTC	1.59 ± 0.51	1.60 ± 0.48	−0.09	1.60 ± 0.56	1.20 ± 0.32	3.68 **

AUTM = autonomy; COMP = competence; RL = relationship; AG = aggressiveness; IBC = irresponsibility and low commitment; DESO = disobedience; PERT = disruptive; AUTC = self-control; (*) = *p* < 0.01; (**) = *p* < 0.001.

## Data Availability

Not applicable.
